# Impact of comorbidities on EQ-5D quality-of-life index in severe asthma

**DOI:** 10.1016/j.jacig.2024.100286

**Published:** 2024-05-31

**Authors:** Paul E. Pfeffer, Thomas Brown, Rekha Chaudhuri, Shoaib Faruqi, Robin Gore, Liam G. Heaney, Adel H. Mansur, Thomas Pantin, Mitesh Patel, Hitasha Rupani, Salman Siddiqui, Aashish Vyas, John Busby, UK Severe Asthma Registry, Martin Doherty, Matthew Masoli

**Affiliations:** a Barts Health NHS Trust, London, United Kingdom; b Queen Mary University of London, London, United Kingdom; c Portsmouth Hospitals University NHS Trust, Portsmouth, United Kingdom; d Gartnavel General Hospital and University of Glasgow, Glasgow, United Kingdom; e Hull University Teaching Hospitals NHS Trust, Hull, United Kingdom; f Addenbrookes Hospital, Cambridge, United Kingdom; g Queen’s University, Belfast, United Kingdom; h University Hospitals Birmingham NHS Trust, Birmingham, United Kingdom; i University of Birmingham, Birmingham, United Kingdom; j Wythenshawe Hospital, Manchester, United Kingdom; k Derriford Hospital, Plymouth, United Kingdom; l University Hospital Southampton NHS Foundation Trust, Southampton, United Kingdom; m National Heart and Lung Institute, Imperial College, London, United Kingdom; n Lancashire Teaching Hospitals NHS Foundation Trust, Preston, United Kingdom; o Centre for Public Health, Queen’s University Belfast, Belfast, United Kingdom

**Keywords:** Corticosteroids, comorbidity, treatable traits, severe asthma

## Abstract

**Background:**

Severe asthma pathology encompasses a wide range of pulmonary and extrapulmonary treatable traits with a high prevalence of comorbidities. Although asthma-specific health-related quality-of-life measures are most sensitive to changes in asthma control, generic measures, such as EQ-5D-5L (EuroQol 5-Dimension 5-Level questionnaire), are potentially better for capturing the impact of comorbidities.

**Objective:**

We sought to examine the impact of pulmonary and extrapulmonary treatable traits on quality of life at initial severe asthma assessment, and to compare the characteristics of those patients whose quality of life does and does not improve during follow-up at severe asthma centers.

**Methods:**

Patients’ characteristics at baseline assessment within the UK Severe Asthma Registry were compared by EQ-5D-5L utility index quartile. Patients with follow-up review data were stratified by change in EQ-5D-5L utility index from baseline to follow-up, and characteristics similarly examined.

**Results:**

Patients in the quartiles with worst dysutility at baseline were observed to exhibit more treatable traits and in particular extrapulmonary traits associated with cumulative systemic corticosteroids, including obesity, anxiety/depression, and osteoporosis. In those patients whose quality of life improved over follow-up, a reduction in exacerbations, uncontrolled symptoms, and requirement for maintenance oral corticosteroids were observed.

**Conclusions:**

Both pulmonary and extrapulmonary treatable traits are important determinants of quality of life in severe asthma. Comorbidities associated with cumulative systemic corticosteroid exposure are particularly associated with worse quality of life, emphasizing the importance of early identification and management of severe asthma before comorbidities develop.

Health-related quality of life is consistently identified by patients with chronic disease as of major importance alongside disease-related mortality. In most long-term conditions, quality of life can be measured with disease-specific and generic (disease- agnostic) quality-of-life measures.^1^^,^^2^ The former are generally more sensitive to small variations in a specific disease’s direct impact on patients, whereas the latter better captures the impact of a disease with its comorbidities on the overall health of a patient.^3^ This is true in asthma, with asthma-specific quality-of-life measures more sensitive to differences in asthma control, but EQ-5D-5L (EuroQol 5-Dimension 5-Level questionnaire), a generic measure, more sensitive to the impact of comorbidities.^4^^,^^5^ EQ-5D-5L is a quality-of-life self-report instrument in which patients rate their health status across 5 dimensions—mobility, self-care, usual activities, pain/discomfort, and anxiety/depression.^6^^,^^7^ It does not measure symptoms directly related to specific pathologies. Generic instruments have an advantage of capturing the impact of disease and comorbidity in a comparable manner across diseases.

Previous studies have shown that patients with asthma on average have worse quality of life than healthy reference populations.^8^^,^^9^ However, severe asthma is a heterogeneous condition in which different pulmonary and extrapulmonary comorbidities and treatable traits are frequently present.^10^ Several extrapulmonary comorbidities in severe asthma, such as osteoporosis and obesity, are particularly a consequence of cumulative oral corticosteroid (OCS) treatment over the course of their asthma history,^11^^,^^12^ and many patients report these as having as much impact on quality of life as asthma itself.^13^ Side effects of corticosteroids are such an issue in severe asthma that the Severe Asthma Questionnaire has been developed to encompass in one questionnaire the impact of both asthma and asthma-related treatment burden on quality of life.^14^

There is increasing interest in the prevention of avoidable extrapulmonary comorbidities in asthma, and their management once present, with severe asthma patient care increasingly extending to include management of both pulmonary and extrapulmonary treatable traits.^15^^,^^16^ Understanding the impact of both pulmonary and extrapulmonary traits on health-related quality of life is therefore of increasing importance.

In this analysis, we first sought to compare the characteristics, in terms of pulmonary and extrapulmonary treatable traits, of patients with severe asthma with least and most impaired quality of life, as assessed by the EQ-5D, at initial severe asthma assessment. Second, we sought to compare the characteristics of those patients whose quality of life does and does not improve during follow-up in specialist severe asthma centers.

## Methods

### Study population

The UK Severe Asthma Registry (UKSAR) collects standardized data on patients with severe asthma who have been referred to specialist services in England, Scotland, and Northern Ireland. Variables contained within the data set include demographic characteristics, patient medical history, current treatment regimes, lung function, and inflammatory biomarkers.^10^ All patients in this analysis had their first clinic visit between January 2015 and October 2021, and were assessed as meeting European Respiratory Society/American Thoracic Society criteria for a severe asthma diagnosis.^17^ Patients were included if they were aged 18 years or older at first assessment, were not receiving biologic therapy at the time of their baseline assessment, and completed the EQ-5D-5L as an assessment of health-related quality of life as part of an initial multidimensional systematic assessment.^10^ Those patients who had also completed the EQ-5D-5L at their first annual review (defined as between 9 months and 24 months after their baseline visit) were included in an analysis of EQ-5D-5L utility change. The UKSAR has database ethical approval from the Office of Research Ethics Northern Ireland (15/NI/0196), and all patients provide written informed consent.

### Exposures, outcomes, and covariates

The EQ-5D-5L contains 5 questions related to the dimensions of mobility, self-care, usual activities, pain/discomfort, and anxiety/depression. For each dimension, patients select 1 of 5 severity levels of perceived impairment/difficulty, generating a possible 3125 health states for each individual patient. A perceived health “value” is applied to each health state on the basis of value sets from the general population, with a valued utility index of 1.0 representing health-related quality of life in a patient with full health and values less than 0.0 representing health-related quality of life perceived as being worse than being dead. For this analysis, we used a previously published value set for England.^7^

We identified a list of treatable traits from the previous literature,^18^^,^^19^ and available variables in UKSAR,^10^ and separated these into pulmonary (uncontrolled asthma symptoms, exacerbations, hospital admission(s), eosinophilia, elevated fractional exhaled nitric oxide [Feno], reduced FEV_1_) and extrapulmonary (atopy, obesity, nasal polyps, depression/anxiety, osteoporosis, gastroesophageal reflux, current smoking). The traits were further categorized into OCS-related (obesity, depression/anxiety, osteoporosis, gastroesophageal reflux) and T2-related (eosinophilia, elevated Feno, atopy) traits. For the pulmonary traits, the presence of uncontrolled asthma symptoms was defined as a 6-item Asthma Control Questionnaire score greater than 1.5,^20^ exacerbations as 2 or more courses of rescue steroids in the previous year, eosinophilia as a blood eosinophil count greater than 300 cells/μL, elevated Feno as a Feno measure in excess of 50 parts per billion, and reduced FEV_1_ as a measure below the lower limit of normal according to GLI 2012 reference equations. For the nonpulmonary traits, atopy was defined on the basis of physician evaluation of patient-reported atopic comorbidities, and obesity as a body mass index in excess of 30 kg/m^2^. Full details on each trait are provided in Table E1 (in the Online Repository available at www.jaci-global.org).

### Statistical analyses

Descriptive statistics were calculated using means ± SD, medians (interquartile ranges), and counts (percentages) as appropriate. We categorized baseline EQ-5D-5L utility, and change in EQ-5D-5L utility from baseline, into similar-sized quartiles ranging from lowest to highest utility. Exactly equal-sized quartiles were not possible due to multiple patients reporting the same utility at the quartile boundaries. Patient demographics, medication, pulmonary traits, and extrapulmonary traits were compared by quartile, and differences between groups were tested for statistical significance using χ^2^ tests, ANOVA, and Kruskal-Wallis tests as appropriate. Ordered logit models were used to assess whether treatable traits mediated ethnic and sex disparities in the EQ-5D-5L. Separate models were fitted for ethnicity and sex, and included hospital site as a fixed effect alongside each of the 13 pulmonary and nonpulmonary traits. All analyses were conducted under a complete-case framework using STATA 16 (StataCorp, College Station, Tex).

## Results

### Characteristics of patients with severe asthma with least and most impaired quality of life at initial severe asthma assessment

The study inclusion criteria were met by 2191 patients who reported a broad range of EQ-5D-5L responses. Impairment was evident across all 5 dimensions, though the median level of impairment was greatest for Mobility, Usual Activities, and Pain/Discomfort across quartiles, stratified by EQ-5D-5L utility (see Table E2 in this article’s Online Repository at www.jaci-global.org).

The quartile with highest EQ-5D-5L utility index (best quality of life) had a median utility index of 0.95, close to that associated with full health (Table I). However, in the quartile with lowest utility index (worst quality of life), the median index was 0.34, signifying extremely poor quality of life.

**Table I:** Baseline characteristics of UK patients with severe asthma quartile stratified by baseline EQ-5D-5L utility index of quality of life

Characteristics	Entire cohort	1 (lowest EQ-5D)	2	3	4 (highest EQ-5D)	*P* value
**No. of patients (N = 2191)**	2191	545	497	583	566	
**EQ-5D-5L Utility (N = 2191)**	0.75 (0.62-0.89)	0.34 (0.17-0.49)	0.69 (0.65-0.71)	0.81 (0.78-0.83)	0.95 (0.92-1.00)	<.001
**EuroQol VAS score (N = 1804)**	60 (40-74)	40 (30-55)	49 (30-60)	60 (45-70)	75 (65-85)	<.001
**Demographics**						
Age at first assessment (y) (N = 2190)	49.9 ± 15.0	50.6 ± 14.0	50.0 ± 14.5	49.6 ± 15.1	49.6 ± 16.3	.644
Age of onset (y) (N = 1989)						.783
<12	703 (35.3)	163 (33.3)	170 (37.7)	194 (36.5)	176 (34.1)	
12-18	214 (10.8)	54 (11.0)	50 (11.1)	57 (10.7)	53 (10.3)	
>18	1072 (53.9)	273 (55.7)	231 (51.2)	281 (52.8)	287 (55.6)	
Sex (N = 2191)						<.001
Female	1,388 (63.4)	400 (73.4)	329 (66.2)	362 (62.1)	297 (52.5)	
Male	803 (36.6)	145 (26.6)	168 (33.8)	221 (37.9)	269 (47.5)	
Ethnicity (N = 2164)						<.001
White	1,836 (84.8)	438 (81.4)	402 (81.5)	499 (87.1)	497 (88.8)	
Non-White	328 (15.2)	100 (18.6)	91 (18.5)	74 (12.9)	63 (11.3)	
**Medication**						
Maintenance OCS (N = 2140)	875 (40.9)	233 (44.0)	197 (40.0)	213 (37.5)	232 (42.3)	.146
Maintenance OCS (mg) (N = 853)	8 (0-10)	10 (10-20)	5 (0-10)	5 (0-10)	6 (0-10)	<.001
ICS Dose (BDP equivalent μg) (N = 2018)	2000 (1600-2000)	2000 (1600-2000)	2000 (1600-2000)	2000 (1200-2000)	2000 (1600-2000)	.157
LAMA (N = 2120)	1221 (57.6)	316 (60.2)	304 (62.3)	343 (60.5)	258 (47.8)	<.001
Theophylline (N = 2114)	563 (26.6)	144 (27.8)	158 (32.2)	171 (30.3)	90 (16.7)	<.001
Leukotriene receptor antagonists (N = 2110)	1315 (62.3)	334 (63.9)	325 (67.1)	351 (62.5)	305 (56.4)	.004
Maintenance macrolides (N = 2062)	246 (11.9)	74 (14.5)	59 (12.4)	72 (13.1)	41 (7.8)	.006
Nebulizer (N = 2091)	492 (23.5)	163 (31.4)	144 (29.8)	121 (21.7)	64 (12.1)	<.001
**Pulmonary traits**						
Asthma symptoms (N = 2092)	1762 (84.2)	509 (97.7)	459 (96.6)	495 (88.6)	299 (55.7)	<.001
2 or more exacerbations (N = 2092)	1743 (83.3)	462 (87.8)	401 (85.9)	467 (83.8)	413 (76.2)	<.001
Hospital admissions (N = 2129)	852 (40.0)	249 (47.0)	222 (45.7)	210 (37.2)	171 (31.2)	<.001
Eosinophilia (N = 2070)	1000 (48.3)	246 (46.2)	212 (45.5)	256 (47.3)	286 (54.0)	.024
Elevated Feno (ppb) (N = 1925)	660 (34.3)	149 (30.4)	131 (31.1)	170 (33.5)	210 (41.4)	<.001
FEV_1_ below LLN (N = 2034)	1279 (62.9)	360 (71.4)	309 (66.2)	336 (62.0)	274 (52.6)	<.001
**Extrapulmonary traits**						
Atopic disease (N = 2069)	1245 (60.2)	315 (60.2)	288 (62.7)	324 (59.0)	318 (59.1)	.609
Obesity (N = 2122)	1058 (49.9)	332 (63.4)	287 (58.9)	267 (46.9)	172 (31.7)	<.001
Nasal polyps (N = 2191)	284 (13.0)	52 (9.5)	58 (11.7)	75 (12.9)	99 (17.5)	<.001
Depression/Anxiety (N = 2191)	348 (15.9)	114 (20.9)	115 (23.1)	88 (15.1)	31 (5.5)	<.001
Osteoporosis (N = 2191)	92 (4.2)	33 (6.1)	19 (3.8)	27 (4.6)	13 (2.3)	.017
GORD (N = 2191)	712 (32.5)	150 (27.5)	203 (40.8)	215 (36.9)	144 (25.4)	<.001
Current smoker (N = 2170)	116 (5.3)	44 (8.2)	33 (6.6)	29 (5.0)	10 (1.8)	<.001

Quartile means ± SD, medians (interquartile ranges), and counts (percentages) as appropriate.

*BDP*, Beclomethasone dipropionate; *GORD*, gastroesophageal reflux disease; *ICS*, inhaled corticosteroid; *LAMA*, long-acting muscarinic antagonist; *LLN*, lower limit of normal; *ppb*, parts per billion; *VAS*, visual analogue scale (quality-of-life score).

The proportion of patients of female sex significantly differed across quartiles (*P* < .001), with the proportion increasing across quartiles from 52.5% in the highest EQ-5D-5L quartile to 73.4% in the lowest EQ-5D-5L quartile. The proportion of patients of non-White ethnicity similarly increased from highest to lowest EQ-5D-5L quartile. The proportion of patients on maintenance OCS did not significantly differ across quartiles, but for those on maintenance OCS, the daily dose did significantly differ (*P* < .001), with the highest dose in the lowest EQ-5D-5L quartile. The proportions of patients on long-acting muscarinic antagonist inhalers, theophyllines, leukotriene receptor antagonists, maintenance macrolides, and nebulizers significantly differed across quartiles, but only for nebulizer usage was there a clear progression across quartiles, with increasing usage from highest (12.1%) to lowest EQ-5D-5L quartile (31.4%).

The proportions of patients with uncontrolled asthma symptoms, with multiple exacerbations in the last year, and hospital admissions with acute asthma in the last year all significantly increased from highest to lowest EQ-5D-5L quartile. In contrast, the proportion with elevated Feno decreased significantly from highest to lowest EQ-5D-5L quartile, with similar pattern for proportion of patients with eosinophilia.

Among extrapulmonary treatable traits, the proportion of patients with each OCS-related comorbidity generally increased from highest to lowest EQ-5D-5L quartile, with, for example, the proportion with osteoporosis rising from 2.3% to 6.1%, and for obesity from 31.7% to 63.4%. In contrast, the proportion with nasal polyps, a comorbidity associated with steroid-sensitive eosinophilic airways disease, decreased from highest to lowest EQ-5D-5L quartile, and the proportion with atopy did not significantly differ across quartiles.

To assess whether the significant associations between demographic factors and EQ-5D-5L quality-of-life quartiles could be mediated by differences in individual treatable traits, we analyzed ordered logit models. The associations between female sex and non-White (minority group) ethnicity and proportion of patients in worse quality-of-life quartile were attenuated after adjustment for treatable traits (see Fig E1 in this article’s Online Repository at www.jaci-global.org).

**Fig 1:**
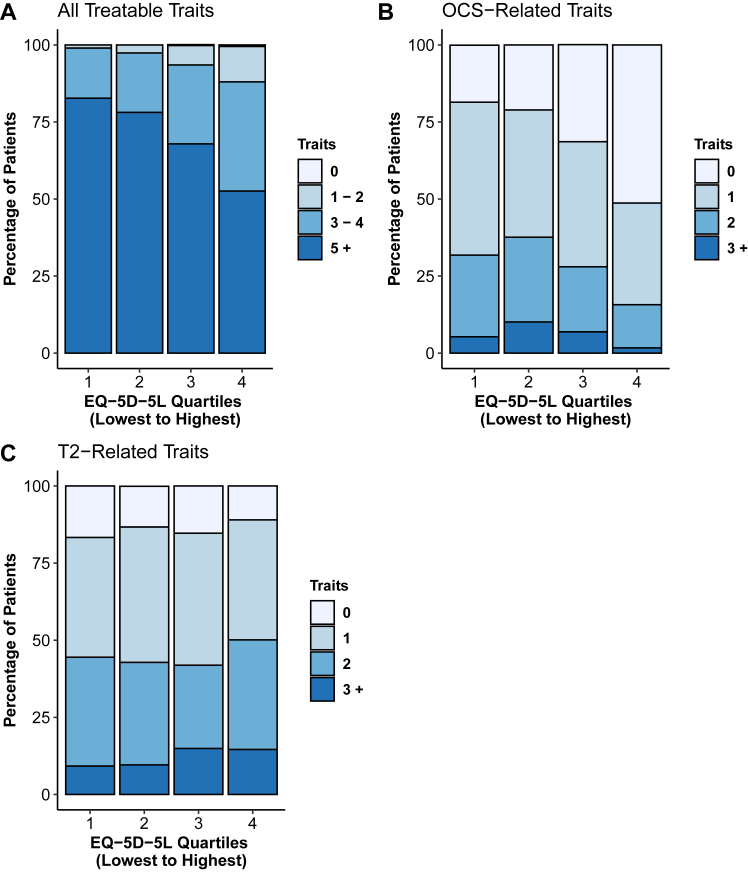
Stacked bar charts of percentages of patients with different number of treatable traits evident at baseline within each quartile of baseline health-related quality of life. **A,** Treatable traits, **B,** OCS-related traits, **C,** T2-related traits. Quartiles graphed from quartile 1 (lowest EQ-5D-5L utility index signifying most impaired quality of life) to quartile 4 (highest EQ-5D-5L utility index signifying least impaired quality of life).

Combining the 13 pulmonary and extrapulmonary treatable traits, the total number of treatable traits per patient significantly differed across quartiles, with a higher number of treatable traits evident in patients in the quartile of lowest EQ-5D-5L quality of life (Fig 1, *A*; see Table E3 in this article’s Online Repository at www.jaci-global.org). Examining OCS-related treatable traits, a similarly significant difference across quartiles was seen, with decreasing OCS-related traits evident in those patients with better quality of life. For example, 51.3% of patients in the quartile with highest EQ-5D-5L quality of life had no OCS-related treatable traits compared with 18.5% in the lowest EQ-5D-5L quartile (Fig 1, *B*). However, with T2-related treatable traits, no clear progression in number of T2-related traits per patient was evident comparing from the quartile with lowest EQ-5D-5L to highest EQ-5D-5L (Fig 1, *C*).

### Characteristics of patients with severe asthma whose quality of life improves or worsens over follow-up in a severe asthma center

Follow-up data with EQ-5D-5L utility index were available from 515 patients. There was a considerable range of change in EQ-5D-5L from baseline to follow-up. After quartile stratification, at one extreme was a quartile with median change in EQ-5D-5L of −0.16 (interquartile range, −0.25 to −0.11), signifying deterioration in quality of life over the period, whereas at the other extreme, there was a quartile with mean change of +0.24 (interquartile range, 0.18 to 0.38) in EQ-5D-5L, signifying large improvement in quality of life (Table II).

**Table II:** Asthma control at follow-up in patients quartile stratified by change in EQ-5D-5L utility index of quality of life from baseline to follow-up

Characteristics	Entire cohort	1 (largest EQ-5D deterioration)	2	3	4 (largest EQ-5D improvement)	*P* value
No. of patients (N = 515)	515	127	130	128	130	
Difference EQ-5D-5L utility (baseline to follow-up) (N = 515)	0.00 (−0.06 to 0.13)	−0.16 (−0.25 to −0.11)	0.00 (−0.03 to 0.00)	0.06 (0.04 to 0.09)	0.24 (0.18 to 0.38)	<.001
Difference EuroQoL VAS score (baseline to follow-up) (N = 355)	0 (−21 to 15)	−10 (−34 to 5)	−3 (−28 to 10)	2 (−15 to 15)	20 (0 to 35)	<.001
Exacerbations (last year) (N = 504)	1 (0 to 4)	2 (0 to 5)	1 (0 to 4)	1 (0 to 4)	1 (0 to 3)	.012
0	191 (37.9)	33 (26.6)	55 (43.7)	44 (35.5)	59 (45.4)	
1	71 (14.1)	21 (16.9)	13 (10.3)	19 (15.3)	18 (13.8)	
2	58 (11.5)	14 (11.3)	10 (7.9)	21 (16.9)	13 (10.0)	
3	41 (8.1)	12 (9.7)	10 (7.9)	7 (5.6)	12 (9.2)	
4+	143 (28.4)	44 (35.5)	38 (30.2)	33 (26.6)	28 (21.5)	
Exacerbation reduction>50% or no exacerbations (N = 494)	339 (68.6)	74 (60.2)	78 (63.4)	84 (69.4)	103 (81.1)	.002
Hospital admissions (last year) (N = 510)	95 (18.6)	36 (29.0)	21 (16.3)	18 (14.2)	20 (15.4)	.008
Uncontrolled asthma symptoms (N = 474)	289 (61.0)	92 (78.6)	64 (54.2)	73 (60.8)	60 (50.4)	<.001
Difference ACQ-6 score (N = 457)	−0.7 (−1.5 to 0.0)	−0.1 (−0.8 to 0.5)	−0.5 (−1.0 to 0.2)	−0.8 (−1.7 to −0.2)	−1.3 (−2.5 to −0.7)	<.001
ACQ score improvement ≥0.5 or controlled (N = 457)	287 (62.8)	47 (42.0)	67 (59.3)	77 (65.3)	96 (84.2)	<.001
FEV_1_ (mL) (N = 435)	2196.7 ± 822.2	2100.3 ± 828.1	2278.5 ± 867.8	2181.2 ± 810.4	2220.6 ± 780.8	.451
FEV_1_ increase >100 mL (N = 429)	161 (37.5)	31 (31.0)	32 (29.6)	45 (39.8)	53 (49.1)	.011
Maintenance OCS (N = 511)	214 (41.9)	65 (51.6)	56 (43.8)	46 (35.9)	47 (36.4)	.037
OCS Change (N = 252)						.048
Discontinue	63 (25.0)	8 (13.1)	22 (29.7)	16 (28.1)	17 (28.3)	
Decreased dose	120 (47.6)	32 (52.5)	32 (43.2)	24 (42.1)	32 (53.3)	
Unchanged dose	48 (19.0)	12 (19.7)	12 (16.2)	16 (28.1)	8 (13.3)	
Increased dose	21 (8.3)	9 (14.8)	8 (10.8)	1 (1.8)	3 (5.0)	
Biologic therapy at follow-up (N = 512)	348 (68.0)	84 (66.1)	80 (62.5)	82 (64.1)	102 (79.1)	.017

Quartile means ± SD, medians (interquartile ranges), and counts (percentages) as appropriate.

*ACQ*, Asthma Control Questionnaire; *ACQ-6*, 6-item Asthma Control Questionnaire; *VAS*, visual analogue scale (quality-of-life score).

**Fig 2:**
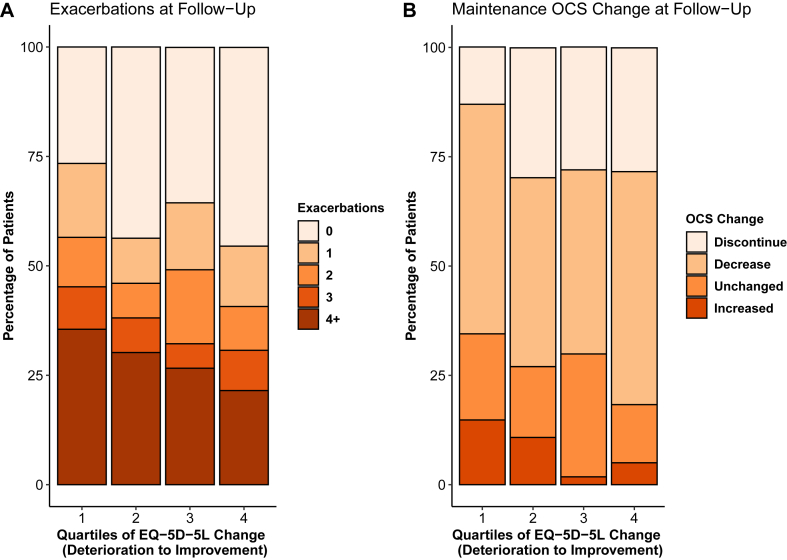
Stacked bar charts of patient characteristics at follow-up within each quartile of change in health-related quality of life. Within-quartile percentages of patients characterized by (**A**) number of exacerbations in preceding year at follow-up review and (**B**) change in maintenance OCS use from baseline to follow-up review. Quartiles graphed from quartile 1 (largest deterioration in EQ-5D-5L utility index signifying greatest decrease in quality of life from baseline to follow-up) to quartile 4 (largest increase in EQ-5D-5L utility index, greatest increase in quality of life).

There was a significantly increasing proportion of patients with 50% or greater reduction in exacerbations or no exacerbations at follow-up (81.1% vs 60.2%), a decreasing proportion with uncontrolled asthma symptoms (50.4 vs 78.6%), a decreasing proportion on maintenance OCSs at follow-up (36.4% vs 51.6%), and an increasing proportion with increase in FEV_1_ of 100 mL or more (49.1% vs 31.0%) among those with the greatest improvement in the EQ-5D-5L when compared with those with the greatest deterioration (Table II and Fig 2).

Although the proportion of patients on biologic therapy at follow-up was highest in the quartile of greatest improvement in EQ-5D-5L from baseline to follow-up, the proportions were similar across the other 3 quartiles (Table II). In a further analysis of change in EQ-5D-5L restricted to those on biologic therapy at follow-up (Table III), there were significant differences across quartiles in the proportions of patients having a clinical response to biologics and being in clinical remission at follow-up. In the quartile with deteriorating EQ-5D-5L, the proportion of patients responding to biologics (78.8%) and being in remission at follow-up (9.9%) was much lower than other quartiles (response and remission respectively 94.0% and 33.3% in the quartile with greatest improvement in EQ-5D-5L).

**Table III:** Response to biologics at follow-up in patients on biologics, quartile stratified by change in EQ-5D-5L utility index of quality of life from baseline to follow-up

Characteristics	Entire cohort	1 (largest EQ-5D deterioration)	2	3	4 (largest EQ-5D improvement)	*P* value
No. of patients (N = 348)	348	84	80	82	102	348
Difference in EQ-5D-5L utility (baseline to follow-up) (N = 348)	0.03 (−0.06 to 0.16)	−0.18 (−0.25 to −0.11)	0.00 (−0.03 to 0.00)	0.07 (0.05 to 0.10)	0.25 (0.19 to 0.39)	<.001
Biological therapy (N = 344)						.061
Omalizumab	26 (7.6)	3 (3.7)	4 (5.1)	8 (9.9)	11 (10.8)	
Dupilumab	1 (0.3)	1 (1.2)	0 (0.0)	0 (0.0)	0 (0.0)	
Mepolizumab	234 (68.0)	61 (74.4)	61 (77.2)	52 (64.2)	60 (58.8)	
Benralizumab	81 (23.5)	17 (20.7)	14 (17.7)	19 (23.5)	31 (30.4)	
Reslizumab	2 (0.6)	0 (0.0)	0 (0.0)	2 (2.5)	0 (0.0)	
Clinical response (N = 338)	293 (86.7)	63 (78.8)	65 (84.4)	71 (87.7)	94 (94.0)	.024
Clinical remission (N = 302)	76 (25.2)	7 (9.9)	23 (33.8)	16 (21.9)	30 (33.3)	.002

Quartile means ± SD, medians (interquartile ranges), and counts (percentages) as appropriate. Clinical response to biologics was defined as a ≥50% reduction in exacerbations and/or maintenance OCS daily dose with no new maintenance OCS initiation. Clinical remission on biologics was defined as requiring a follow-up ACQ-5 score <1.5, no exacerbations, and maintenance OCS daily dose of ≤5 mg prednisolone-equivalent.

When assessing quartile-stratified quality of life in patients on biologics at follow-up assessment, there was a significant difference in the proportion of patients in clinical remission at follow-up across quartiles (from 14.8% in the quartile with lowest EQ-5D-5L index to 40.3% in the quartile with highest EQ-5D-5L index), though not in the proportion achieving clinical response (see Table E4 in this article’s Online Repository at www.jaci-global.org).

Given the higher prevalence of extrapulmonary treatable traits in patients with worse quality of life at baseline, we next examined whether these traits at baseline were associated with relative improvement or deterioration in quality of life over the follow-up period (Table IV). There was significant variation in the prevalence of obesity across the quartiles of EQ-5D-5L change, with higher baseline prevalence of obesity in patients in the quartiles with greater EQ-5D-5L improvement, although there was no evidence of a dose-response relationship. The prevalence of other extrapulmonary traits did not significantly differ across quartiles of EQ-5D-5L change.

**Table IV:** Prevalence of baseline extrapulmonary traits in patients quartile stratified by improvement in EQ-5D-5L utility index of quality of life from baseline to follow-up

Characteristics	Entire cohort	1 (largest EQ-5D deterioration)	2	3	4 (largest EQ-5D improvement)	*P* value
**No. of patients (N = 515)**	515	127	130	128	130	
**Baseline EQ-5D-5L utility index (N = 515)**	0.74 (0.53 to 0.89)	0.75 (0.51 to 0.92)	0.88 (0.71 to 1.00)	0.75 (0.65 to 0.87)	0.51 (0.22 to 0.74)	<.001
**Baseline EuroQoL VAS score (N = 405)**	60 (45 to 75)	59 (40 to 70)	70 (50 to 80)	60 (45 to 75)	50 (40 to 69)	<.001
**Demographics**						
Age at first assessment (y) (N = 515)	50.9 ± 13.9	51.9 ± 14.1	49.9 ± 13.4	50.5 ± 13.0	51.2 ± 15.1	.677
Sex (N = 515)						.462
Female	315 (61.2)	76 (59.8)	73 (56.2)	82 (64.1)	84 (64.6)	
Male	200 (38.8)	51 (40.2)	57 (43.8)	46 (35.9)	46 (35.4)	
Ethnicity (N = 515)						.889
White	436 (84.7)	105 (82.7)	112 (86.2)	109 (85.2)	110 (84.6)	
Non-White	79 (15.3)	22 (17.3)	18 (13.8)	19 (14.8)	20 (15.4)	
**Extrapulmonary traits**						
Atopic disease (N = 2069)	299 (59.7)	77 (62.1)	70 (56.5)	76 (60.8)	76 (59.4)	.823
Obesity (N = 2122)	250 (48.9)	63 (49.6)	46 (36.2)	70 (55.1)	71 (54.6)	.008
Nasal polyps (N = 2191)	83 (16.1)	22 (17.3)	21 (16.2)	22 (17.2)	18 (13.8)	.863
Depression/Anxiety (N = 2191)	88 (17.1)	25 (19.7)	18 (13.8)	19 (14.8)	26 (20.0)	.423
Osteoporosis (N = 2191)	28 (5.4)	12 (9.4)	5 (3.8)	5 (3.9)	6 (4.6)	.147
GORD (N = 2191)	131 (25.4)	34 (26.8)	30 (23.1)	41 (32.0)	26 (20.0)	.141
Current smoker (N = 2170)	29 (5.7)	4 (3.1)	4 (3.2)	9 (7.0)	12 (9.2)	.090

Quartile means ± SD, medians (interquartile ranges), and counts (percentages) as appropriate.

*GORD*, Gastroesophageal reflux disease; *VAS*, visual analogue scale (quality-of-life score).

## Discussion

The baseline-stratified data show increasing uncontrolled asthma symptoms and exacerbations among those with the worse quality of life. Furthermore, the patients with worst quality of life at baseline had the highest rates of comorbidities associated with cumulative systemic corticosteroid toxicity. However, the T2 biomarkers of elevated Feno and blood eosinophilia, which would be expected to rise with increasing exacerbation risk and increasing steroid responsiveness, decreased among those with worse quality of life such that there is growing discordance between biomarker profile and exacerbation burden in this patient group. Improvement in quality of life over follow-up was associated with a reduction in exacerbations and maintenance oral steroids, reduction in uncontrolled asthma symptoms, and improvement in lung function. Consistent with this, the proportion of patients with severe asthma in clinical remission on biologics was highest in those patients with best quality of life at follow-up. Prevalence of extrapulmonary traits at baseline, including OCS-related comorbidities, were not in general significantly different between those patients whose quality of life improved or deteriorated over follow-up.

Our findings are consistent with the published literature, for example with associations between female sex and impaired health-related quality of life,^9^ and worse quality of life in patients with uncontrolled asthma.^21^ However, we extend previous findings, showing that associations between demographic factors and impaired quality of life in severe asthma are attenuated by adjustment for treatable traits. Use of EQ-5D-5L allows comparison of our results to studies in other chronic diseases.

Higher prevalence of corticosteroid-associated comorbidities in patients with worse quality of life in our study is consistent with the findings of Sullivan et al^22^ who reported lower EQ-5D health utility in patients who received 4 or more courses of systemic corticosteroids in a year even after adjustment for underlying chronic conditions. Lanario et al^23^ have reported, using the Severe Asthma Questionnaire (SAQ) instrument, that in patients with severe asthma, symptoms of uncontrolled asthma and general (extrapulmonary) symptoms contribute approximately equally to variation in quality-of-life scores and also found patients with high cumulative oral steroid burden to have worse quality of life.^23^ McDonald et al^18^ have examined the relationship between treatable traits in severe asthma and quality of life assessed with the St George’s Respiratory Questionnaire. Similar to us, they found patients with a greater number of treatable traits to have worse quality of life. We observed in those patients with best quality of life that most of the different asthma medication classes were being taken at lower frequency, consistent with the known association between polypharmacy and worse health-related quality of life.^24^ In particular, home nebulizer usage was associated with impaired quality of life.

Lin et al^25^ have recently adopted an inductive approach to clustering patients with severe asthma and traits to generate trait profiles, and found patients with non–airway-centric trait profiles (with higher levels of obesity and other nonairway comorbidities) to have worse quality of life and lower prevalence of eosinophilia. At 6 months following systematic assessment, all trait profiles showed improvement in quality of life, consistent with our finding that baseline comorbidity does not preclude quality-of-life improvement with high-quality severe asthma management.^25^

A broad spread of change in EQ-5D-5L health utility was evident at follow-up, including deterioration in some patients. This is notable given our previous research showing that both patients commencing and not commencing a biologic have improved asthma control and reduced exacerbations at 1-year follow-up after specialist assessment at UK severe asthma centers in UKSAR.^26^ The wide range of change in EQ-5D score from baseline to follow-up is, however, not dissimilar to that observed in other conditions such as after initiation of biologics for rheumatoid arthritis or 1 year following breast cancer treatment with curative intent.^27^^,^^28^

However, EQ-5D-5L has previously been shown to be relatively insensitive to change (improvement) after initiation of biologic therapy in severe asthma,^5^ potentially because EQ-5D-5L does not directly assess common respiratory symptoms such as breathlessness and cough. Nevertheless, those characteristics by which successful response to biologics is assessed (reduction in exacerbation frequency, maintenance oral steroid use, and uncontrolled asthma symptoms) were associated with improvement in EQ-5D-5L utility over the follow-up period. However, addition of a sixth dimension assessing limitation due to breathing problems has been suggested as a means to improve the questionnaire for use in respiratory diseases such as severe asthma.^29^

The wide range in EQ-5D-5L scores for cohort quartiles in this study is similar to the range in EQ-5D-5L seen in other chronic diseases. Furthermore, the median index of 0.34 for the most severe quartile is similar to the lower limits for the ranges of EQ-5D-5L for cancer and cardiovascular disease.^30^ A limitation of UKSAR is that the duration of uncontrolled severe asthma, that is the cumulative years requiring systemic corticosteroids to manage uncontrolled asthma preceding baseline assessment, is not recorded. However, the high incidence in patients with worst quality of life at baseline of comorbidities associated with cumulative systemic corticosteroid exposure suggests that a longer duration of uncontrolled severe asthma leads to more impaired quality of life, with a dysutility in these patients with severe asthma similar to that in cancer. A concern that followed was whether patients with those comorbidities might not receive as much benefit from interventions severe asthma centers can provide—that much of the damage cannot be undone. It is therefore reassuring that there were no clear associations between baseline corticosteroid-related extrapulmonary traits and EQ-5D-5L response over follow-up at a severe asthma center, though this potentially reflects the holistic care provided to patients by UK severe asthma centers that are commissioned to provide multidisciplinary care to patients including physiotherapy, psychology, and, in some centers, dietetic support.^26^

The discordance between type 2 (T2) biomarker profile and both exacerbation burden and comorbidities associated with corticosteroid toxicity in those patients with severe asthma with worst quality of life has several potential explanations. One explanation may be patients who were T2-high but who are now overtreated with corticosteroids, suppressing the T2 biomarkers but leading to complications of systemic steroid treatment including infection-driven exacerbations. Another explanation is that many of the symptoms of these patients are due to pathologies other than the reversible small airways inflammation of asthma, for example, breathing pattern disorders and inducible laryngeal obstruction.^31^^,^^32^ These pathologies do not respond to corticosteroids, and wrong diagnosis leads to the direct complications of inappropriate treatments and also the adverse psychological impact of the lived experience of not responding to treatment.^32^

The high prevalence of steroid-related morbidity in patients with severe asthma at initial assessment at severe asthma centers, with adverse impact on quality of life, could potentially be reduced by earlier referral of patients to specialist centers, and adoption of the treatable traits approach to asthma in primary care.^19^ Cumulative OCS exposure would likely be reduced and comorbidities addressed earlier, reducing their impact.

There is growing interest into the degree to which steroid-related and other extrapulmonary traits can be “reversed” in the biologics era. For example, Nanzer et al^33^ have recently shown that significant weight loss is possible in obese patients after initiation of biologics. Although our research does suggest that prevention of these comorbidities, with earlier access to biologics before comorbidities develop, would improve quality of life across severe asthma populations, it does not address whether treating these extrapulmonary traits once developed improves quality of life and further research is urgently needed.

The strengths of this research reflect the strengths of UKSAR and in particular the approach to systematic assessment of asthma now undertaken as standard across the UK collaborating centers. UKSAR follows international consensus guidance on data fields to collect.^34^ Although this ensures a feasible scale of data collection, it does result in a paucity of data on some clinical characteristics that would have been of interest for this study, such as assessments for breathing pattern disorders and inducible laryngeal obstruction. An ever-increasing number of treatable traits in asthma are being described in research publications, including traits not captured in large registries. For instance, Janssen et al^35^ recently reported that worse quality of life in asthma is associated with greater number of treatable traits, frequent exacerbations, and obesity, similar to our findings, but also found fatigue, a novel asthma trait, to potentially have the most impact on quality of life. Obstructive sleep apnea and sleep quality are other important traits in severe asthma, affecting asthma control and quality of life, though not systematically recorded as traits in UKSAR.^36^^,^^37^ A limitation of registry research is that new data fields cannot easily be retrospectively collected as new variables become germane, as our understanding of severe asthma develops. Linkage from disease registries to primary care records could provide rich data sets in which to explore the impact of diverse treatable traits further. Although current research shows the impact of treatable traits and comorbidities on quality of life in severe asthma, it does not answer the question of the relative importance of different traits in determining quality of life or potential interactions between treatable traits. Larger linked data sets with repeated measures over time would allow approaches such as causative inference modeling to address such future research aims.

In summary, patients with severe asthma with worst quality of life are characterized by high exacerbation burdens but discordantly low T2 biomarkers, and have a quality of life similar to patients with other common, major conditions such as cancers. Presence of comorbidities associated with cumulative systemic corticosteroid exposure is associated with worse quality of life, emphasizing the importance of reaching these patients and improving their asthma management early before these comorbidities develop.

**Clinical implications**

It is important to diagnose severe asthma and commence biologics before patients develop comorbidities associated with systemic corticosteroids given their impact on quality of life, but patients who have those comorbidities still benefit from specialist treatments.

## Disclosure statement

Disclosure of potential conflict of interest: P. E. Pfeffer has attended advisory board for 10.13039/100004325AstraZeneca (AZ), 10.13039/100004330GlaxoSmithKline (GSK), and Sanofi; has given lectures/webinars at meetings with/without lecture honoraria supported by AZ, Chiesi, and GSK; has attended international conferences with AZ, GSK, 10.13039/100004336Novartis, and Sanofi; has taken part in clinical trials sponsored by AZ, GSK, Novartis, 10.13039/100009857Regeneron, and Sanofi; and is conducting research funded by GSK for which his institution receives remuneration. T. Brown has received speaker fees from GSK, AZ, Teva, Chiesi, and Sanofi; honoraria for advisory board meetings from AZ, Sanofi, and Teva; sponsorship to attend international scientific meetings from Chiesi, Teva, Novartis, and Sanofi; and fees as an external expert for the AZ Pathway Programme. R. Chaudhuri has received lecture fees from GSK, AZ, Teva, Chiesi, Sanofi, and Novartis; honoraria for advisory board meetings from GSK, AZ, and Celltrion; sponsorship to attend international scientific meetings from Chiesi, Sanofi, and GSK; and a research grant to her institute from AZ for a UK multicenter study. S. Faruqi has received speaker fees and sponsorship to attend specialty meetings from AZ, GSK, Chiesi, Novartis, and Sanofi. R. Gore has received lecture fees from GSK, Sanofi, AZ, and Novartis and sponsorship to attend national scientific meetings from Sanofi UK. L. Heaney has received grant funding, participated in advisory boards, and given lectures at meetings supported by Amgen, AZ, Boehringer Ingelheim, Chiesi, Circassia, Hoffmann la Roche, GSK, Novartis, Theravance, Evelo Biosciences, Sanofi, and Teva; has received grants from MedImmune, Novartis UK, Roche/Genentech, Inc, and GSK, Amgen, Genentech/Hoffman la Roche, AZ, MedImmune, Aerocrine, and Vitalograph; has received sponsorship for attending international scientific meetings from AZ, Boehringer Ingelheim, Chiesi, GSK, and Napp Pharmaceuticals; has also taken part in asthma clinical trials sponsored by AZ, Boehringer Ingelheim, Hoffmann la Roche, and GSK for which his institution received remuneration; is the Academic Lead for the 10.13039/501100000265Medical Research Council Stratified Medicine UK Consortium in Severe Asthma, which involves industrial partnerships with a number of pharmaceutical companies including Amgen, AZ, Boehringer Ingelheim, GSK, Hoffmann la Roche, and Janssen. A. H. Mansur has received funds personal and to institution from AZ, GSK, Novartis, Chiesi, Boehringer Ingelheim, and Sanofi for talks, advisory board meetings, educational grant, and sponsorships to attend conferences. T. Pantin has received advisory board honoraria from AZ, speaker fees from GSK, and sponsorship for attending conferences from GSK, Sanofi, and Chiesi. H. Rupani has received speaker fees from GSK, AZ, Chiesi, Sanofi, and Boehringer Ingelheim; conference support from AZ; grant funding to her institution from AZ and GSK; and has attended advisory boards for AZ and GSK. S. Siddiqui has received advisory board and/or speaker fees from GSK, AZ, Chiesi, Areteia Therapeutics, CSL Behring, ERT Medical, and Medscape; grant funding from AZ; and is a cofounder of Eupnoos Limited. J. Busby reports research grants from AZ and personal fees from Nuvoair. The rest of the authors declare that they have no relevant conflicts of interest.
