# Supplementary material for: Impact of comorbidities on EQ-5D quality-of-life index in severe asthma
Source: J Allergy Clin Immunol Glob. 2024 May 31;3(3):100286. doi: 10.1016/j.jacig.2024.100286 (PMC11277382; doi:10.1016/j.jacig.2024.100286)
Supplement: Supplementary data [file mmc1.docx]

**Impact of comorbidities on EQ5D quality of life index in severe asthma**

**Paul E. Pfeffer, PhD,** Consultant Respiratory Physician, Barts Health NHS Trust, London, UK; and Hon Senior Lecturer, Barts and The London School of Medicine and Dentistry, Queen Mary University of London, UK

**Thomas Brown, PhD,** Consultant Respiratory Physician, Portsmouth Hospitals University NHS Trust, Portsmouth, UK

**Rekha Chaudhuri, MD,** Respiratory Physician, Gartnavel General Hospital and University of Glasgow, Glasgow, UK

**Shoaib Faruqi, MD,** Consultant Respiratory Physician, Hull University Teaching Hospitals NHS Trust, Hull, UK

**Robin Gore, PhD,** Consultant Respiratory Physician, Addenbrookes Hospital, Cambridge, UK

**Liam Heaney, MD,** Professor of Respiratory Medicine, Queen’s University, Belfast, UK

**Adel H Mansur, PhD,** Consultant Respiratory Physician, University Hospitals Birmingham NHS Trust, Birmingham, UK; and Hon Professor, University of Birmingham, Birmingham UK

**Thomas Pantin, MD,** Consultant Respiratory Physician, Wythenshawe Hospital, Manchester, UK

**Mitesh Patel, PhD,** Consultant Respiratory Physician, Derriford Hospital, Plymouth, UK

**Hitasha Rupani**, **PhD,** Consultant Respiratory Physician, University Southampton NHS Foundation Trust, Southampton, UK

**Salman Siddiqui**, **PhD,** Professor of Respiratory Medicine, National Heart and Lung Institute, Imperial College, London UK.

**Aashish Vyas, MD,** Consultant Respiratory Physician, Lancashire Teaching Hospitals NHS Foundation Trust, UK

**John Busby, PhD,** Senior Lecturer in Medical Statistics, Centre for Public Health, Queen’s University Belfast, UK

on behalf of the **UK Severe Asthma Registry**

**Supplemental Data**

**Supplemental Table E1**

| **Trait** | **Description** | **Cut point** |
| --- | --- | --- |
| **PULMONARY TRAITS** |  |  |
| Asthma Symptoms | Asthma symptoms measured by the Asthma Control Questionnaire (ACQ-6) | >1.5 score |
| Exacerbations | Exacerbations in the year prior to the baseline visit | 2 or more |
| Hospital admissions | Hospitalisations for asthma in the year prior to the baseline visit | 1 or more |
| Eosinophilia^b^ | Blood eosinophil count measured at the baseline visit | >300 cells/uL |
| FeNO (ppb)^b^ | Fractional exhaled nitric oxide measured at the baseline visit | >50ppb |
| FEV1 | FEV1 measured at the baseline visit | Below lower limit of normal according the Global Lung Initiative 2012 reference equations |
| **EXTRA-PULMONARY TRAITS** |  |  |
| Atopic Disease^b^ | Patient reported atopic co-morbidity | Yes / No |
| Obesity^a^ | BMI measured at the baseline visit | >30 kg/m^2^ |
| Nasal Polyps | Nasal polyps recorded during the baseline visit | Yes / No |
| Depression / Anxiety^a^ | Depression / Anxiety recorded during the baseline visit | Yes / No |
| Osteoporosis^a^ | Osteoporosis recorded during the baseline visit | Yes / No |
| GORD^a^ | GORD recorded during the baseline visit | Yes / No |
| Current Smoker | Smoking status recorded during the baseline visit | Current Smokers vs. Never / Ex-smoker |

**Supplemental Table I: Definition of traits and rationale for cut points**

^a^ Oral corticosteroid (OCS)-related trait

^b^ T2-biomarker related trait

**Supplemental Table E2**

|  | **Entire Cohort** | **1 (Lowest EQ-5D)** | **2** | **3** | **4 (Highest EQ-5D)** | **P-value** |
| --- | --- | --- | --- | --- | --- | --- |
| **DIMENSION MEDIAN SCORES** |  |  |  |  |  |  |
| **EuroQoL Mobility** | 2.0 (1.0,3.0) | 4.0 (3.0,4.0) | 2.0 (2.0,3.0) | 2.0 (1.0,2.0) | 1.0 (1.0,1.0) | <0.001 |
| **EuroQoL Self-care** | 1.0 (1.0,2.0) | 3.0 (2.0,3.0) | 2.0 (1.0,2.0) | 1.0 (1.0,1.0) | 1.0 (1.0,1.0) | <0.001 |
| **EuroQoL Usual Activities** | 2.0 (2.0,3.0) | 4.0 (3.0,4.0) | 3.0 (2.0,3.0) | 2.0 (2.0,3.0) | 1.0 (1.0,2.0) | <0.001 |
| **EuroQoL Pain or Discomfort** | 2.0 (1.0,3.0) | 4.0 (3.0,4.0) | 3.0 (2.0,3.0) | 2.0 (1.0,2.0) | 1.0 (1.0,1.0) | <0.001 |
| **EuroQoL Anxiety - Depression** | 2.0 (1.0,3.0) | 3.0 (2.0,4.0) | 2.0 (2.0,3.0) | 2.0 (1.0,2.0) | 1.0 (1.0,1.0) | <0.001 |

**Supplemental Table II: Median severity of dysutility in each EQ-5D-5L domain in patients quartile stratified by baseline EQ-5D-5L utility index of quality of life**

**Supplemental Table E3**

|  | **Entire Cohort** | **1 (Lowest EQ-5D)** | **2** | **3** | **4 (Highest EQ-5D)** | **P-value** |
| --- | --- | --- | --- | --- | --- | --- |
| **COMBINED TRAITS** |  |  |  |  |  |  |
| **Number of traits; N=1557** |  |  |  |  |  | <0.001 |
| 0 | 3 (0.2%) | 0 (0.0%) | 0 (0.0%) | 1 (0.3%) | 2 (0.5%) |  |
| 1-2 | 85 (5.5%) | 4 (1.0%) | 9 (2.6%) | 25 (6.3%) | 47 (11.5%) |  |
| 3-4 | 379 (24.3%) | 66 (16.3%) | 67 (19.3%) | 102 (25.6%) | 144 (35.4%) |  |
| 5+ | 1,090 (70.0%) | 334 (82.7%) | 271 (78.1%) | 271 (67.9%) | 214 (52.6%) |  |
| **OCS-RELATED TRAITS** |  |  |  |  |  |  |
| Number of traits; N=2122 |  |  |  |  |  | <0.001 |
| 0 | 657 (31.0%) | 97 (18.5%) | 103 (21.1%) | 179 (31.5%) | 278 (51.3%) |  |
| 1 | 871 (41.0%) | 260 (49.6%) | 201 (41.3%) | 231 (40.6%) | 179 (33.0%) |  |
| 2 | 469 (22.1%) | 139 (26.5%) | 134 (27.5%) | 120 (21.1%) | 76 (14.0%) |  |
| 3+ | 125 (5.9%) | 28 (5.3%) | 49 (10.1%) | 39 (6.9%) | 9 (1.7%) |  |
| **T2-RELATED TRAITS** |  |  |  |  |  |  |
| Number of traits; N=1804 |  |  |  |  |  | 0.002 |
| 0 | 254 (14.1%) | 78 (16.7%) | 52 (13.2%) | 72 (15.3%) | 52 (11.0%) |  |
| 1 | 739 (41.0%) | 181 (38.8%) | 173 (43.9%) | 201 (42.8%) | 184 (38.9%) |  |
| 2 | 591 (32.8%) | 165 (35.3%) | 131 (33.2%) | 127 (27.0%) | 168 (35.5%) |  |
| 3+ | 220 (12.2%) | 43 (9.2%) | 38 (9.6%) | 70 (14.9%) | 69 (14.6%) |  |

**Supplemental Table III: Numbers of treatable traits evident in patients (total, OCS-related and T2-related) quartile stratified by baseline EQ-5D-5L utility index of quality of life**

OCS, oral corticosteroid.

**Supplemental Table E4**

|  | **Entire Cohort** | **1 (Lowest EQ-5D)** | **2** | **3** | **4 (Highest EQ-5D)** | **P-value** |
| --- | --- | --- | --- | --- | --- | --- |
| **Number of Patients; N=360** | 348 | 100 | 74 | 86 | 88 |  |
| **EuroQoL Utility; N=360** | 0.74 (0.47,0.89) | 0.26 (0.13,0.41) | 0.68 (0.62,0.70) | 0.81 (0.78,0.83) | 0.95 (0.92,1.00) | <0.001 |
| **EuroQol VAS Score; N=292** | 60 (50,75) | 45 (30,64) | 55 (45,65) | 60 (50,70) | 75 (70,90) | <0.001 |
| **Clinical Response; N=350** | 293 (86.7%) | 78 (79.6%) | 64 (90.1%) | 76 (89.4%) | 75 (89.3%) | 0.109 |
| **Clinical Remission; N=314** | 76 (25.2%) | 13 (14.8%) | 11 (16.4%) | 23 (30.7%) | 29 (40.3%) | <0.001 |

**Supplemental Table IV: Biologic response at follow-up of UK severe asthma patients, quartile stratified by follow-up EQ-5D-5L utility index of quality of life.**

Quartile means (standard deviation [SD]), medians (interquartile ranges [IQR]) and counts (percentages) as appropriate. OCS, oral corticosteroid; VAS, visual analogue scale (quality of life score). Clinical response to biologics was defined as a ≥50% reduction in exacerbations and/or maintenance OCS daily dose with no new maintenance OCS initiation. Clinical remission on biologics was defined as requiring a follow-up ACQ5<1.5, no exacerbations and maintenance OCS daily dose of ≤ 5mg prednisolone-equivalent.

**Supplemental Figure E1**

**
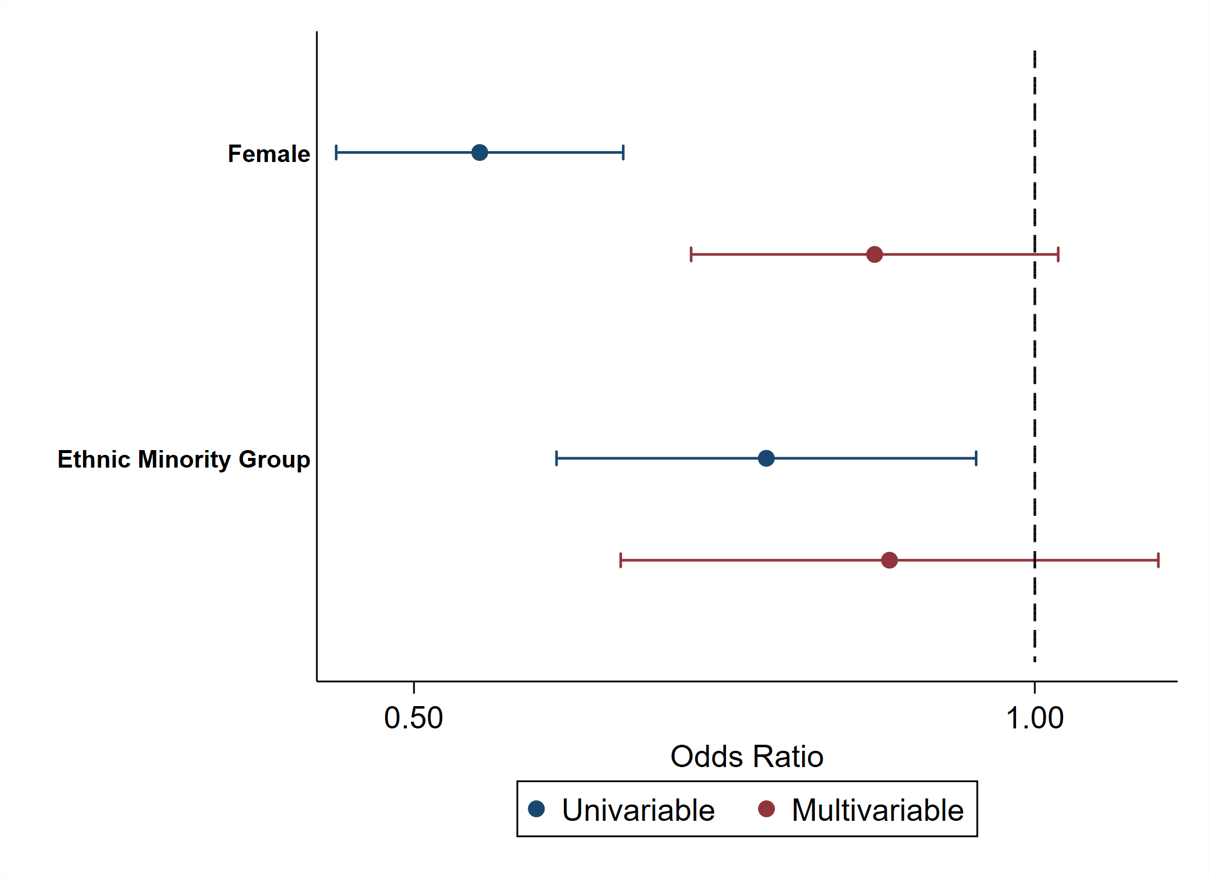
**

**Supplemental Figure 1: Effect of adjusting for treatable traits on associations between gender and ethnicity and EQ-5D-5L quartile.**

Odds Ratio of belonging to a higher EQ5D quartile in unadjusted analysis (univariable) and after adjustment for treatable traits (multivariable). Ordered logit models with separate models fitted for ethnicity and sex, and included hospital site as a fixed-effect alongside each of the 13 pulmonary and non-pulmonary traits.
